# Whole-brain patterns of ^1^H-magnetic resonance spectroscopy imaging in Alzheimer's disease and dementia with Lewy bodies

**DOI:** 10.1038/tp.2016.140

**Published:** 2016-08-30

**Authors:** L Su, A M Blamire, R Watson, J He, L Hayes, J T O'Brien

**Affiliations:** 1 Department of Psychiatry, School of Clinical Medicine, University of Cambridge, Cambridge, UK; 2 Institute of Cellular Medicine, Newcastle Magnetic Resonance Centre, Newcastle University, Newcastle upon Tyne, UK; 3 Department of Aged Care, Royal Melbourne Hospital, Parkville, VIC, Australia; 4 Aberdeen Biomedical Imaging Centre, Aberdeen University, Aberdeen, UK

## Abstract

Magnetic resonance spectroscopy has demonstrated metabolite changes in neurodegenerative disorders such as Alzheimer's disease (AD) and dementia with Lewy bodies (DLB); however, their pattern and relationship to clinical symptoms is unclear. To determine whether the spatial patterns of brain-metabolite changes in AD and DLB are regional or diffused, and to examine whether the key metabolite levels are associated with cognitive and non-cognitive symptoms, we acquired whole-brain spatially resolved 3T magnetic resonance spectroscopic imaging (MRSI) data from subjects with AD (*N*=36), DLB (*N*=35) and similarly aged controls (*N*=35). Voxel-wise measurement of *N*-acetylaspartate to creatine (NAA/Cr), choline to Cr (Cho/Cr), myo-inositol to Cr (mI/Cr) as well as glutamate and glutamine to Cr (Glx/Cr) ratios were determined using MRSI. Compared with controls, AD and DLB groups showed a significant decrease in most brain metabolites, with NAA/Cr, Cho/Cr and mI/Cr levels being reduced in posterior cingulate, thalamus, frontotemporal areas and basal ganglia. The Glx/Cr level was more widely decreased in DLB (posterior cingulate, hippocampus, temporal regions and caudate) than in AD (only in posterior cingulate). DLB was also associated with increased levels of Cho/Cr, NAA/Cr and mI/Cr in occipital regions. Changes in metabolism in the brain were correlated with cognitive and non-cognitive symptoms in the DLB but not in the AD group. The different patterns between AD and DLB may have implications for improving diagnosis, better understanding disease-specific neurobiology and targeting therapeutics. In addition, the study raised important questions about the role of occipital neuroinflammation and glial activation as well as the glutamatergic treatment in DLB.

## Introduction

Alzheimer's disease (AD) and dementia with Lewy bodies (DLB) are the two commonest subtypes of degenerative dementia in older people.^1^ They share common clinical and neuropsychological features. In DLB, there appears to be an interruption of awareness, which is often associated with transient episodes of confusion and communication difficulties. Remission to near-normal cognitive function can occur spontaneously in the absence of clear environmental triggers. In addition to such cognitive fluctuation, visual hallucinations, which are false perceptions in the absence of an external stimulus that are accompanied by a compelling sense of reality, and motor Parkinsonism are also the core clinical features of DLB. In addition, subjects with DLB show poor executive function, attention and visual processing.^2, 3^ Not only basic processing speed but also high-level attention functions, such as selective attention and the sustained attention, are impaired in DLB.^4^ These attentional deficits have an important role in other symptoms of DLB such as visual hallucinations.^5^

Several biophysical systems have been tested in DLB. A putative candidate system is the basal–forebrain cholinergic system, which has widespread projections to the neocortex. In DLB, it is well established that there is a marked loss of basal–forebrain cholinergic neurons compared with AD;^6^ furthermore, abnormality of nicotinic receptor binding in DLB patients appears to be associated with disturbances of consciousness.^7, 8^ Pharmacological evidence has demonstrated that anticholinergic drugs can induce a symptom profile of altered consciousness that is similar to cognitive fluctuation in DLB.^9^ Conversely, cholinesterase inhibitors significantly improve the cognitive fluctuation in DLB.^9^ Cortical systems responsible for arousal and attention may be also affected in DLB, therefore contributing to the development of cognitive fluctuation, for example, dopamine and noradrenergic systems as well as hypothalamus and midbrain that mediate arousal and circadian function.^10, 11^

DLB can be characterised by the presence within neurons of Lewy bodies, containing misfolded α-synuclein protein; however, in comparison with AD, it has less severe brain atrophy, instead with a more diffuse and less focal pattern,^12, 13^ and relatively preserved medial temporal lobes.^14, 15, 16^ In addition, β-amyloid plaques and tau neurofibrillary tangles (that is, AD-like pathologies) are also found in DLB. While lacking regional-specific structural changes, studies of brain metabolism and neurotransmitter systems using metabolic 18-fluoro-deoxyglucose positron emission tomography and perfusion single-photon emission computed tomography have highlighted the involvement of occipital,^17, 18, 19^ temporoparietal^20^ and subcortical areas^21, 22, 23^ in DLB. There is also evidence in AD that such functional imaging changes can be detected many years before structural atrophy and the onset of clinical symptoms.^24^ Thus, studying patterns of brain biochemical changes in AD and DLB can provide important information for diagnosis and treatments as well as for understanding disease-specific neurobiology.

*In vivo* assessment of metabolite levels using ^1^H-magnetic resonance spectroscopy has provided a substantial amount of evidence on biochemical abnormalities in AD and DLB.^25^ It has been reported that the *N*-acetylaspartate (NAA) level was lower in patients with AD in posterior cingulate, frontal, temporal and parietal lobes.^26, 27, 28^ NAA has frequently been seen as a neuronal marker, and was also found to be decreased in several other dementia types, such as DLB.^29, 30^ Regarded as a glia marker, myo-inositol (mI) was found to be elevated in AD^16, 17^ and DLB.^31^ Reduction in glutamate and glutamine (Glx) has also been found in AD^32^ and DLB.^33^ Thus, the literature supports the potential utility of MR spectroscopy to assist ante-mortem detection for AD and DLB.

However, the majority of previous studies have either focused on a single voxel, often not in an area known to be affected by pathology, or included only a very small number of brain regions. This approach is limited in its ability to provide full information about whether changes in AD and DLB are regional or diffused as dysfunction outside these areas cannot be assessed. The literature on MRS in AD and DLB has provided quite disparate results,^16^ which is likely due, at least in part, to differences in the location of targeted brain regions. Finally, because of the low signal-to-noise ratio in MRS, the reported effect size in most previous studies was relatively small, making it very sensitive to the selection of brain regions. Hence, there is a pressing need to provide unbiased and robust voxel-wise whole-brain estimates for MRS data in dementia.

Here, we used a ^1^H-magnetic resonance spectroscopic imaging (MRSI) method to overcome the above-mentioned limitations in previous research in order to investigate the spatial pattern of MRSI in AD, DLB and similarly aged control groups. We also assessed the relationship between MRS changes and clinical and cognitive measurements in subjects with AD and DLB.

## Materials and methods

### Subject recruitment and assessment

The study was performed on 106 subjects in three groups (AD, DLB and cognitively normal controls). Thirty-six individuals with probable AD and thirty-five people with probable DLB were recruited from referrals to secondary care old-age psychiatry and neurology services.^34^ Thirty-five controls were recruited from among spouses and friends of the subjects with dementia or from advertisements in local community newsletters. The sample size was selected after a power calculation based on prior literature. For example, Kantarci *et al.*^25^ investigated MRS changes in AD and MCI as well as controls at 1.5 T using single-voxel methods. The mean (s.d.) NAA to creatine (Cr) ratios were 1.53 (0.11) in controls (*N*=85), 1.49 (0.09) in MCI (*N*=49) and 1.43 (0.1) in AD (*N*=60), a reduction of 1 s.d. between AD and controls. Thus, a sample size of *N*=35 in each group gives a power of over 90% to detect a similar magnitude of difference between groups with alpha=0.05 in the current study. All participants underwent clinical and neuropsychological evaluations with diagnostic procedures previously employed by our group.^24^

Assessment of global cognitive function in all participants involved use of the Cambridge Cognitive Examination (CAMCOG), which incorporates the Mini-Mental State Examination (MMSE).^35^ Motor Parkinsonism was assessed with the Unified Parkinson's Disease Rating Scale III (UPDRS).^36^ For subjects with DLB, cognitive fluctuation was evaluated with the Clinician Assessment of Fluctuation Scale.^37^ The research was approved by the local ethics committee. All participants (or carers if subjects lacked capacity) provided written informed consent/assent.

### MRI/MRSI data acquisition

**Figure 1:**
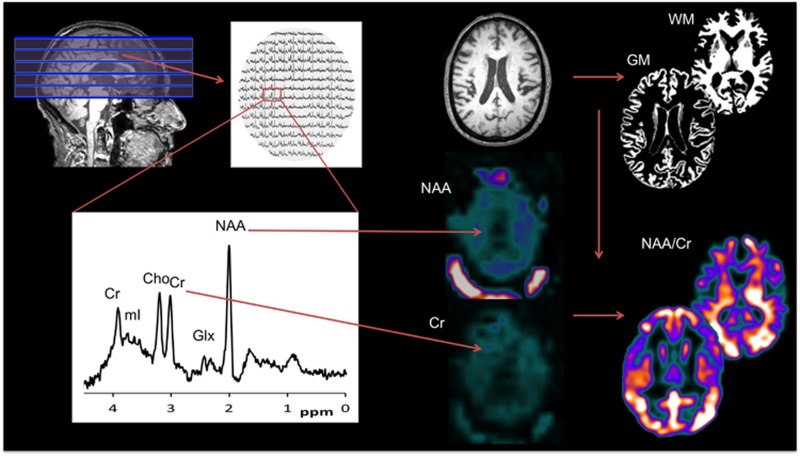
Whole-brain MRS analysis. Each spectral was analysed to extract concentrations of five major metabolites (Cr, Cho, Glx, NAA and mI), which were then converted into 3D maps in subjects' native space. A modified SPM procedure was applied to remove non-brain areas from the metabolism maps and normalise to the standard MNI space using high-resolution T1-weighted MRI data. The ratio between other metabolites and the internal reference Cr was computed before the maps were smoothed for statistical analysis. 3D, 3 dimensional; Cho, choline; Cr, creatine; Glx, glutamate and glutamine; GM, grey matter; mI, myo-inositol; MRI, magnetic resonance imaging; MRS, magnetic resonance spectroscopy; NAA, *N*-acetylaspartate; WM, white matter.

Participants underwent magnetic resonance imaging (MRI) scanning on a 3T Philips Achieva MRI system (Intera Achieva scanner, Philips Medical Systems, Eindhoven, Netherlands) with an eight-channel receiver head coil. Structural images were acquired using a T1-weighted volumetric sequence (3D MPRAGE, sagittal acquisition aligned with the AC-PC line, 1 mm isotropic resolution, matrix 240 × 240 × 180, TR=9.6 ms, TE=4.6 ms, flip angle=8°, SENSE factor=2). MR spectroscopy images were acquired using a multislice, single-spin echo, spectroscopic imaging sequence, incorporating octagonal spatial presaturation bands placed over the skull/scalp to minimise lipid contamination (10 × 10 mm in-plane resolution, five 18 mm slices, matrix 24 × 20 × 5, TR=3450 ms, TE=35 ms). The pack of MRSI slices was angulated along the AC-PC line and covered 90 mm of brain tissue in the superior–inferior direction (Figure 1). Two subjects with AD and one control did not complete the imaging protocol and so were excluded.

### Statistical tests of demographic, clinical and cognitive measures

Group characteristics were evaluated with Statistical Toolbox of Matlab (www.mathworks.co.uk/products/statistics). Differences in demographic and clinical data were assessed with use of either *t*-tests for continuous variables or *χ*^2^-tests for categorical measures. For each test statistic, a probability value of *P*<0.05 was regarded as significant.

### Mass spectral analysis

Within each voxel of MRSI, we analysed the spectra using jMRUI v.4 (http://www.mrui.uab.es/mrui/) to quantify the level of NAA, Cho, mI, Glx and creatine (Cr). Residual water was removed using an SVD filter with 20 components,^38^ and the initial spectral zero-order phasing was manually adjusted across all voxels. Then, we used the QUEST algorithm^39^ to quantify each metabolite based on a predefined metabolite database, which captured the spectral profiles of these metabolites *in vitro*. During fitting, zero-order phase variation was constrained to be ±45 Hz of the initial estimate. The results of QUEST analysis were a set of estimated levels for all five metabolites in each voxel for each subject. Instead of analysing a small number of spectral for each subject, we performed a mass analysis for all 2400 voxels within a single subject scan and for all 104 subjects.

### MRI segmentation and normalisation

Volumetric structural MRI (T1-weighted) images were first segmented using Gaussian mixture model implemented in the VBM toolbox, and brain tissues were classified into grey matter (GM), white matter (WM) and cerebrospinal fluid. GM probability maps were nonlinearly normalised to standard MNI space (www.mni.mcgill.ca) using the diffeomorphic registration algorithm (DARTEL) in SPM v.8.^40^

### MRSI and MRI co-registration

MRS images have a lower resolution (10 × 10 × 18 mm) than the structural T1 images (1 × 1 × 1 mm); therefore, linear interpolation was applied to MRSI data before co-registering them to the MRI data using a modified Gannet v.2 function (gabamrs.blogspot.co.uk). Owing to the lack of anatomical information in the MRSI data, conventional registration methods (for example, mutual information approach) are not applicable. Instead, the Gannet-based approach assumes that the MRSI and accompanying structural scan are in good registration and uses the image header information (image origin coordinates, angulation and voxel size) to match MRSI and MRI data. The quality of co-registration was validated by visual inspection for any motion-related mis-co-registration. That is, we overlaid the averaged MRS maps of all metabolites including a map of lipid signal on the anatomical MRI from the same subject and visually checked whether the key anatomical landmarks (lateral ventricles and scalp) are registered correctly because cerebrospinal fluid has a low level of metabolism and the scalp has a high level of lipid signal.

### MRS image normalisation and smoothing

After co-registeration, we masked MRS maps of GM and WM by tissue probability maps derived from T1-weighted MRI images in each participant's native space in order to remove non-brain areas (*c.f.* Figure 1). We then transformed the masked MRS maps into the standard MNI space using the participant-specific diffeomorphic parameters estimated from the previous DARTEL procedure. However, we did not apply modulation in order to avoid confound of age and disease-related brain volume changes, and preserve the original biochemical concentration in the patients' space. We then computed the images of the ratios between the metabolites of interest (NAA, Cho, mI and Glx) and the internal reference (Cr). In order to reduce the noise level, we further smoothed the MRS maps with an isotropic Gaussian kernel of 8 mm full-width at half-maximum.

### Considerations for partial volume effect

The use of ratio between brain metabolite and the internal reference is common in most MRS studies in the literature. Here, it corrected for the spatial variation in the sensitivity profile of the head coil but also, more importantly for the potential partial volume effect caused by non-brain tissue. Specifically, in areas of atrophy, which are expected in these patient groups, tissue loss may reduce the levels of all metabolites, but particularly Cr, which is ubiquitous to all cells and hence take the ratio to Cr, should ameliorate this potential confound of partial volume.

### Whole-brain voxel-wise analysis

In order to explore disease-related differences in the metabolic profile in AD, DLB and control subjects, we used the General Linear Model with age and gender as covariates, and then performed ‘omnibus' tests of 3 groups × 4 metabolites factorial analysis of variance (ANOVA) for the main effect of groups in GM and WM separately. The investigators were blinded to the clinical diagnosis before this stage of the analysis. The false-positive rate was controlled using the standard family-wise error correction for multiple comparisons implemented in SPM statistical package, and was thresholded at *P*<0.05 at the cluster level.

### *Post hoc* regional analysis

On the basis of the whole-brain analysis, we extracted the averaged metabolite signal levels from significant clusters. *Post hoc* *t*-tests were performed in order to explore which metabolite or group was driving the results in the whole-brain ANOVA. We also correlated the averaged signal from each cluster with cognitive and clinical measures including the CAMCOG, UPDRS, MMSE and Clinician Assessment of Fluctuation scale. The *post hoc* analysis was uncorrected for multiple comparisons because it was based on the whole-brain family-wise error-corrected clusters.

## Results

### Demographic, clinical and cognitive measures

There were no significant differences among AD, DLB and control groups for age, sex and education (Table 1). On all cognitive and clinical measures, AD and DLB subjects scored less well than controls (statistics not shown) as we have reported previously in this specific patient cohort.^41, 42^ As expected, subjects with DLB scored significantly higher in UPDRS and cognitive fluctuation scale than AD; however, they did not differ in MMSE and CAMCOG scores.

### Whole-brain ANOVA for the main effect of groups

**Figure 2:**
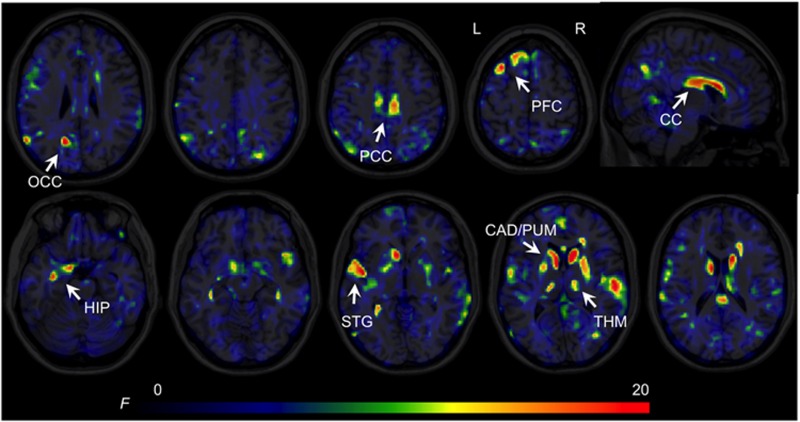
Magnetic resonance spectroscopic imaging (MRSI) statistical parameter (F ratio) maps from whole-brain ANOVA for the main effect of groups. Significant clusters FWE, *P*<0.05) shown in yellow/red are located in both cortical (prefrontal, temporal and cingulate) and subcortical (CAD, PUM and THM) areas. For display purposes, the GM and WM results are shown in different views (axial, GM and sagittal, WM). ANOVA, analysis of variance; CAD, caudate; CC, corpus callosum; FWE, family-wise error; GM, grey matter; HIP, hippocampus, parahippocampus and amygdala; OCC, occipital cortex; PCC, posterior cingulate cortex; PFC, prefrontal cortex; PUM, putamen; STG, superior temporal cortex; THM, thalamus; WM, white matter.

We found a significant main effect of group in the GM of the occipital cortex, posterior cingulate, prefrontal cortex, anterior hippocampus (extending to parahippocampus and amygdala), posterior hippocampus, superior temporal areas, putamen, caudate and thalamus across all metabolites (Figure 2). In the WM, we found a significant cluster in the corpus callosum. (See Supplementary Information for additional group analysis of each metabolite.)

### *Post hoc* analysis within significant clusters

**Figure 3:**
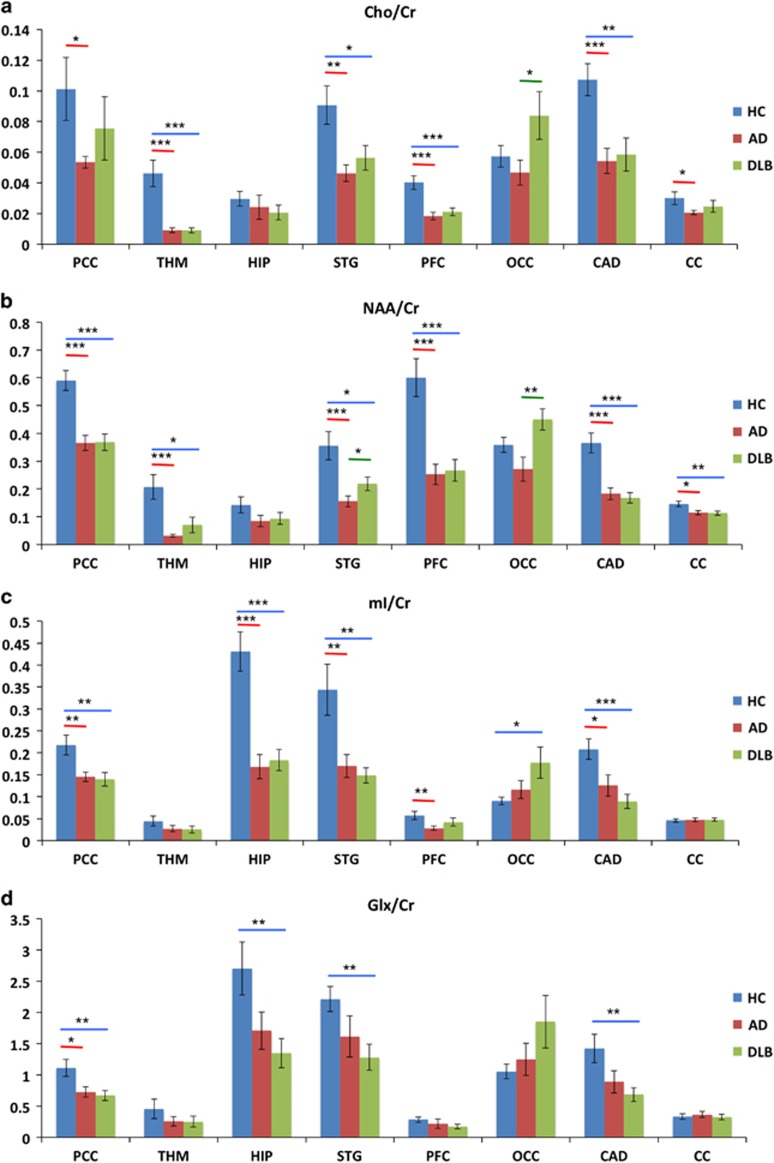
*Post hoc* analyses of significant Anchor clusters from SPM: (**a**) Cho/Cr ratio, (**b**) NAA/Cr ratio, (**c**) mI/Cr ratio and (**d**) Glx/Cr ratio. Horizontal lines represent significant difference (*P*<0.05 uncorrected) between two groups: red, AD versus controls; blue, DLB versus controls; and green, DLB versus AD. Error bars are s.e.m. *P<0.05, **P<0.01 and ***P<0.001. AD, Alzheimer's disease; CAD, caudate; CC, corpus callosum; DLB, dementia with Lewy bodies; HC, controls; HIP, hippocampus, parahippocampus and amygdala; OCC, occipital cortex; PCC, posterior cingulate cortex; PFC, prefrontal cortex; STG, superior temporal cortex; THM, thalamus.

In the *post hoc* analysis, we focused on main clusters derived from the whole-brain ANOVA showing that the main effect of group was mainly driven by reductions of metabolites in AD and DLB relative to their controls (Figure 3).

Subjects with AD and DLB had a significantly reduced Cho/Cr level in the posterior cingulate, thalamus, superior temporal areas, prefrontal cortex and caudate (Figure 3). However, the occipital Cho/Cr level was significantly greater in DLB than in AD. Decreases in NAA/Cr were found in the posterior cingulate, thalamus, superior temporal regions, prefrontal cortex and caudate in AD and DLB. However, subjects with AD had significantly lower NAA/Cr levels in the temporal and occipital cortex than DLB. In AD and DLB, the mI/Cr level was reduced in the posterior cingulate, hippocampus, superior temporal lobes and caudate. In AD, the mI/Cr level was also lower than controls in the prefrontal cortex, whereas in DLB it was greater than controls in the occipital cortex. Subjects with DLB showed a reduction in the Glx/Cr level in the posterior cingulate, hippocampus, temporal cortex and caudate. However, we only found a significant decrease in Glx/Cr in the posterior cingulate in AD, but not in other regions. Finally, in the corpus callosum, we found a reduced Cho/Cr and NAA/Cr levels in AD and a reduced NAA/Cr in DLB.

### Correlation between cognitive/clinical measures and MRSI

**Figure 4:**
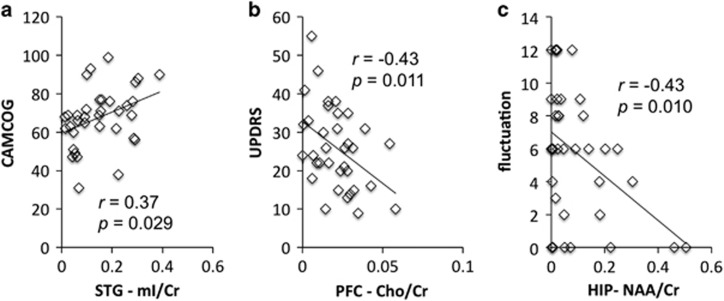
Correlational Anchor analysis with: (**a**) cognition (CAMCOG), (**b**) motor symptoms (UPDRS) and (**c**) cognitive fluctuation. Significant correlation between metabolism and cognitive/clinical measurements (*P*<0.05, uncorrected) in DLB. Averaged levels of different metabolites were extracted from the corresponding significant clusters in the SPM analysis. DLB, dementia with Lewy bodies.

Figure 4 shows that the mI/Cr level was significantly correlated with the CAMCOG score in the temporal cortex in DLB. In the prefrontal cortex, the Cho/Cr was significantly inversely correlated with the UPDRS score in DLB. Finally, the NAA/Cr level in the hippocampus was significantly inversely correlated with the clinician fluctuation scale. We found no significant correlations between MRS data and cognitive/clinical measurements in AD.

## Discussion

Using a novel approach, we investigated whole-brain patterns of brain metabolites using ^1^H-proton MRS in AD and DLB, the two commonest neurodegenerative dementias in older people. Our results showed areas of the brain, in which there were maximum variation in metabolites among the groups in a whole-brain ANOVA. These brain areas involve both cortical and subcortical structures, and in regions where AD and DLB pathology are commonly found.^43^ A cluster occupying middle and posterior cingulate was significant in distinguishing the patient groups from controls in most metabolites studied here, partly supporting findings in the prior literature.^15^ Although both groups showed a pattern of reduction in NAA/Cr, Cho/Cr and mI/Cr levels, the Glx/Cr level was more widely disrupted in DLB than in AD. Our results also suggest that the occipital region is one of the key structures that significantly differentiated the AD and DLB groups. Next, we will discuss findings on each metabolite in turn.

### The Cho/Cr level

In MRS, the Cho signal is mainly because of cytosolic glycerophosphocholine and phosphocholine found in myelin and the cell membrane. Therefore, variation in the Cho signal implies changes in WM integrity and membrane turnover.^44, 45^ Although previous studies showed mixed and inconsistent results,^16^ we found a general reduction in the Cho/Cr level but an increase in occipital regions in DLB. The increase in the occipital Cho/Cr level in DLB compared with AD is a different pattern from the overall reduction of Cho/Cr in the rest of regions of interest. It is possible that membrane (phospholipids) turnover was higher in this brain region in DLB, consistent with previous single-photon emission computed tomography studies showing occipital involvement in DLB^7^ and the visual spatial impairments in DLB. However, it has also been reported that the occipital Cr level might be lower in DLB compared with AD.^18^ This can also result in a higher Cho/Cr ratio. Finally, the Cho/Cr level in the corpus callosum was reduced in AD.

In addition, a reduction in prefrontal Cho/Cr level was correlated with more severe Parkinsonism, that is, the total UPDRS ratings, in DLB. Although it is well known that prefrontal dopamine deficits in Parkinson's disease is strongly related to motor symptoms, and non-dopamine systems mainly contribute to cognitive symptoms,^9^ our data showed that cholinergic activity maybe another predictor for motor symptoms in DLB. This finding also points out the potential interaction between dopaminergic and cholinergic circuitries in prefrontal areas in Lewy body diseases.

### The NAA/Cr level

NAA is mainly located in the bodies, axons and dendrites of neurons; therefore, it has been regarded as a marker of neuronal number, density and viability as well as mitochondrial function.^15^ A reduction in the NAA/Cr level has been consistently reported in neurodegeneration.^16^ Here, we also showed a widespread reduction of the NAA/Cr level in both AD and DLB groups, implicating impairments in neuronal integrity and/or neuronal loss.

In superior temporal and occipital areas, there was a significantly lower NAA/Cr in AD than in DLB, suggesting a more extensive loss of neuronal function in AD. The corpus callosum NAA/Cr level was lower in AD and DLB; however, we did not find a significant reduction in the NAA/Cr level in hippocampus, a brain structure that is first and substantially affected by AD pathology in the same cohort^31^ and in other studies. However, in the DLB group, the level of hippocampal NAA/Cr significantly correlated with the severity of cognitive fluctuation, that is, a lower level of NAA/Cr was linked to higher degree of fluctuation. A very wide range of brain systems has been implicated in cognitive fluctuation,^46^ including attention and default mode network, and hippocampus function is closely coupled with default mode network (for example, posterior cingulate) during task and rest.^47^

### The mI/Cr level

mI is present in glial cells, and thus has been frequently associated with inflammation or gliosis in the brain. Unlike previous studies,^16^ we found an overall decrease of mI/Cr in AD and DLB, implying a possible lower rather than greater extent of glial cell activation in the majority of brain areas. However, the DLB group showed a higher occipital mI/Cr level. In addition, temporal mI/Cr was correlated with cognitive performance (CAMCOG) in the DLB group, reflecting a potential modulatory effect immune reaction in dementia.

Here, in posterior cingulate, superior temporal lobe, hippocampus and basal ganglia structures, we not only saw a reduction in NAA/Cr, Cho/Cr and Glx/Cr, suggesting a decrease in neuronal density and integrity as well as energy metabolism, but also a decrease in glial activation and inflammation reaction. One explanation for our findings is that the inflammation may occur in very early stages of pathology, and, as the disease progresses, this process may become exhausted because of possible glial cell loss. Our cohort, although relatively mild from a clinical viewpoint, could be seen as reasonably advanced in terms of their disease progression as all had a clinical diagnosis of dementia. This, as well as occipital increases of mI/Cr in DLB, which may reflect a heightened inflammatory response there, needs further investigation. Finally, in the occipital region, which is more affected in DLB than in AD, we found increases in both NAA/Cr and Cho/Cr levels, implying a possible compensatory mechanism coexisting with the raised brain immune systems.

### The Glx/Cr level

Glutamate and glutamine metabolism is related to both neurons and glial cells. Separation of signals from glutamate from glutamine is challenging, and so both metabolites are commonly considered together. Reduction in the Glx/Cr level may also imply dysfunction and loss of glutamatergic neurons.^23^ Consistent with previous findings, we showed a widespread decrease in Glx/Cr ratio in DLB, and only in posterior cingulate in AD, suggesting that the widespread Glx/Cr deficit is a distinctive feature in DLB. Of interest, memantine, an N-methyl-D-aspartate antagonist, has been reported to show efficacy in DLB and AD.^48^ Should our results have wider confirmation, this would support the further development and testing of glutamatergic therapies in DLB.

### Comparison with previous studies and design considerations

When compared with GM atrophy,^31^ diffusion tensor imaging (DTI) abnormalities^32^ and relaxometry changes^24^ in the same cohort, we found that the spatial distribution of metabolic dysfunction is more similar to relaxometry and DTI changes than atrophy. In particular, we found significant metabolic changes in subcortical regions, which showed a relative structural preservation in DLB, implicating that MRS and DTI maybe more sensitive in detecting changes in neuronal and glial integrity before there is cell loss.^49^

Our study has advanced from most previous MRS investigations in neurodegenerative diseases, which have only focused on a single or limited number of brain areas, such as posterior cingulate. A significant drawback of such single voxel (or a small number of voxels) approach is *selection bias*, that is, a tendency for new studies to base their hypothesis on existing data and select regions of interest that have already been tested and the null hypothesis rejected. This approach tends to find positive results confirming the prior research but suffers from the risk of a higher type I error rate. A related problem is publication bias,^50^ which favours positive findings over negative ones, thus inflating the real effect size. Therefore, limitations of previous hypothesis-driven methods are as follows: (1) the majority of the brain areas are underexplored and (2) the effect size can be overestimated. For diseases with diffuse patterns of pathology, such as DLB, the previous single-voxel approach is arguably suboptimal. Finally, it is difficult to compare across studies that have different selections of brain region.

In our study, the design and analysis were based on classic statistical analysis because our work was looking for disease-related differences in metabolic profile in AD and DLB. An alternative analysis would be to use the data to perform classification between subject groups using methods such as support vector machine. The emerging machine-learning methods have been used for differentiating AD, MCI and other types of dementia.^51^ Although majority of the imaging studies employed group-level analysis, support vector machine and similar classification techniques can potentially have higher sensitivity and specificity for diagnosis at an individual subject level.^52^ The spatial information about metabolic profile is important to understand the disease-specific neurobiology and relate it to known pathology in these conditions. Therefore, recent development in multivariate analysis of neuroimaging data has advanced from classifying whole-brain pattern to localising regional effects. For example, the ‘searchlight' algorithm, developed by Kriegeskorte and co-workers^53, 54^ for MRI and Su *et al.*^55^ for MEG, allows support vector machine to use regional multivariate pattern information and remain spatially resolved. Thus, future studies, especially those with small sample size or intended for individual-level discrimination, should consider advanced multivariate pattern analyses.

## Conclusion

In summary, our findings on major brain metabolite changes and their correlations with cognitive and non-cognitive symptoms are broadly in agreement with previous research. However, the study used an unbiased whole-brain voxel-wise approach. Our results also raised important questions about the role of occipital neuroinflammation and glial activation as well as the glutamatergic treatment in DLB.

The current study has some limitations. For example, the mass spectral analysis was semi-automatic. In addition, we computed the ratios between metabolites rather than the absolute concentration of metabolites. This approach is sensitive to potential changes in Cr levels, and also makes the interpretation of the results more difficult. Future research should avoid referencing to the Cr level, although the use of such ratio data can naturally address the partial volume effect, which is often caused by severe atrophy in our cohort, without using explicit partial volume correction methods.

## Figures and Tables

**Table 1:** Demographics, clinical and neuropsychological measures

	*Controls*	*DLB*	*AD*	*Statistics*
*N*	34	35	35	—
Gender (m:f)	20:14	27:8	20:15	0.12
Age (years)	76.8 (5.2)	78.4 (6.9)	78.3 (5.8)	0.94
Education (years)	11.6 (2.5)	10.8 (2.6)	11.0 (3.4)	0.06
UPDRS	1.9 (1.9)	26.0 (10.7)	5.6 (4.4)	<0.001
CogFluct	—	6.1 (3.8)	2.0 (3.6)	<0.001
MMSE	29.1 (1.0)	20.3 (5.3)	19.3 (4.3)	0.39
CAMCOG	97.3 (3.8)	67.7 (15.2)	65.3 (11.8)	0.46

Abbreviations: AD, Alzheimer's disease; CAMCOG, Cambridge Cognitive Examination; DLB, dementia with Lewy bodies; f, female; m, male; MMSE, Mini-Mental State Examination; UPDRS, Unified Parkinson's Disease Rating Scale III.

Values expressed as mean (s.d.).

## Supplementary Material

Supplementary Figure A1
