# Supplementary figures and images for: Whole-brain patterns of 1H-magnetic resonance spectroscopy imaging in Alzheimer's disease and dementia with Lewy bodies
Source: Transl Psychiatry. 2016 Aug 30;6(8):e877–. doi: 10.1038/tp.2016.140 (PMC5022086; doi:10.1038/tp.2016.140)

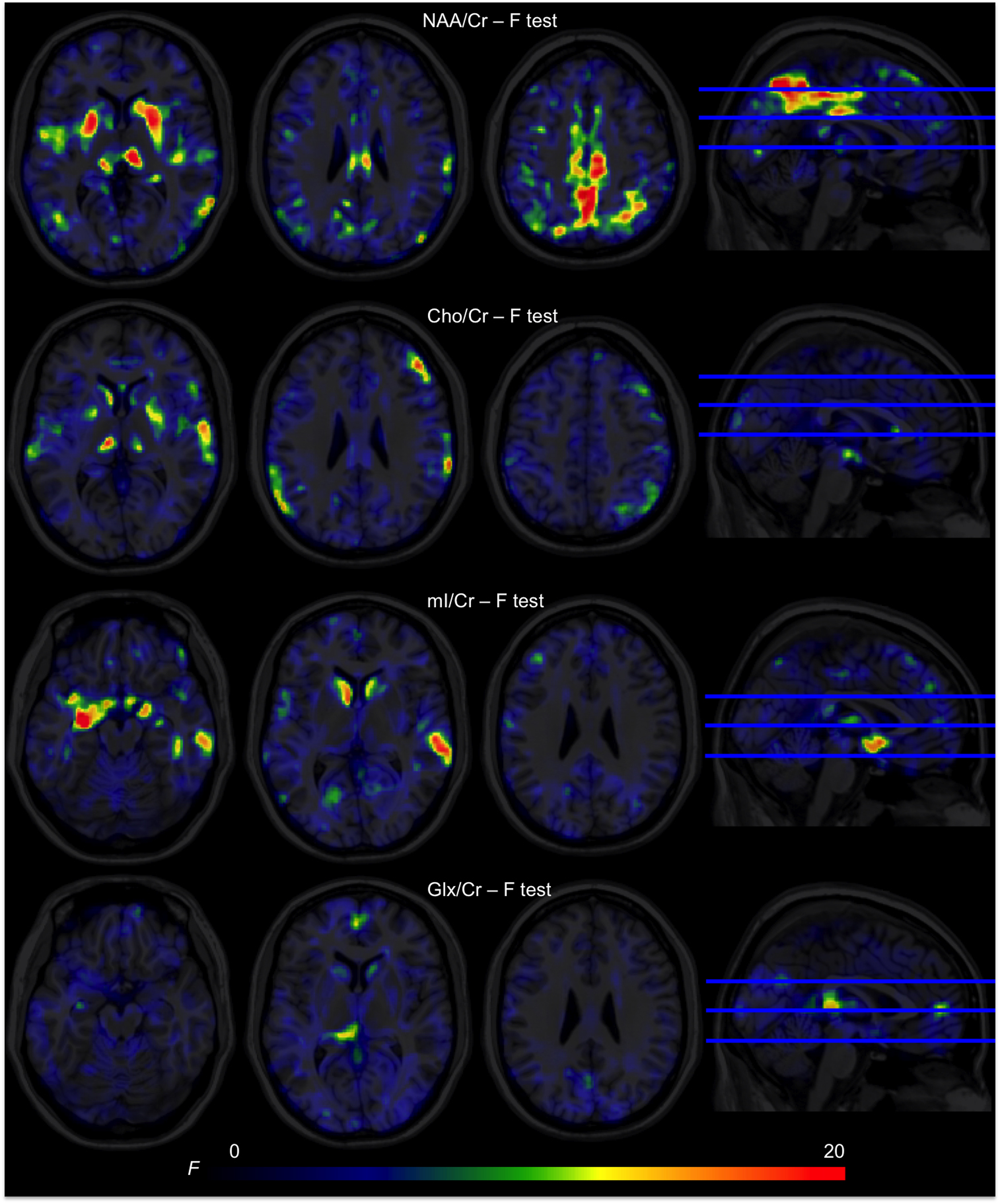

Supplement: Supplementary Figure A1 [file tp2016140x1.tif]
