# Supplementary material for: Whole-brain patterns of 1H-magnetic resonance spectroscopy imaging in Alzheimer's disease and dementia with Lewy bodies
Source: Transl Psychiatry. 2016 Aug 30;6(8):e877–. doi: 10.1038/tp.2016.140 (PMC5022086; doi:10.1038/tp.2016.140)
Supplement: Supplementary Information [file tp2016140x2.doc]

**Supplementary Information:**

**Whole-brain group analysis for each metabolite**

In addition to the “omnibus” test of all metabolites in a combined factorial ANOVA, we also tested the group effect of each metabolite in separate simple *F*-tests. In order to simplify the analysis, we combined the GM and WM during the analysis of individual metabolite. The results are shown in Fig. A.1.

Insert Figure A.1 here

We found no significant effect at alpha level of p < 0.05 after correction for multiple comparisons (FWE) in each of the metabolic ratios (Cho/Cr, NAA/Cr, mI/Cr and Glx/Cr). Using an uncorrected threshold p < 0.001, we found several significantly clusters in posterior cingulate, basal ganglia, thalamus and hippocampus areas (Fig. A.1), which are consistent with the spatial location of group effect in the combined factorial ANOVA (Fig. 2) to some extent. These results confirm the findings reported in the main text.

However, it is notable that the simple *F*-tests were less sensitive than the factorial ANOVA combining all four metabolic ratios in general and particularly for Glx. It is possible that that the noise variance in some metabolites (e.g. Glx) was larger than others because the MRS signal for these metabolites are generally weaker and their characteristic frequencies are too close to other metabolites that have stronger signal. So, in simple *F*-tests, the within group summed squares (or noise variance) was large in Glx, resulting a small *F* ratio given the same between group variance. However, in the factorial ANOVA combining all metabolites, the relatively weaker signal in Glx was supplemented by the strong signal from other metabolites (e.g. NAA) in the same voxel, allowing it to pass the threshold in the omnibus test. In the post-hoc regional analysis, we then test if there was indeed a significant group difference in Glx at these significant clusters.

We argue that the omnibus approach combining all metabolites is preferable in this whole-brain MRSI study, because: 1) it directly pointed us to areas in the brain that there have the largest variation in the overall metabolism implicating areas that may be affected by the diseases, a significant advantage to single voxel approaches used in previous researches; and 2) the concentrations of different metabolites are not statistically independent, thus the omnibus test avoids the need for correcting of multiple comparisons by analyzing all metabolites at once. The correlational structure between different metabolites may come from two sources. Firstly, MRS signals of many brain metabolites share common biochemical sources, e.g. between NAA and Glx. Secondly, the estimated concentrations of these metabolites were derived from the same MR spectra, thus, unavoidable share a common noise structure.

**Figure A.1** **Statistical parameter (F ratio) maps from whole-brain ANOVA for the main effect of groups in each metabolite ratio**. Cho/Cr, NAA/Cr, mI/Cr and Glx/Cr (p < 0.001 uncorrected).
